# Association of energy source with outcomes in en bloc TURB: secondary analysis of a randomized trial

**DOI:** 10.1007/s00345-025-05565-w

**Published:** 2025-03-27

**Authors:** Stefano Mancon, Francesco Soria, Rodolfo Hurle, Dmitry Enikeev, Evanguelos Xylinas, Lukas Lusuardi, Axel Heidenreich, Paolo Gontero, Eva Compérat, Shahrokh F. Shariat, David D’Andrea

**Affiliations:** 1 Department of Urology, Comprehensive Cancer Center, Medical University of Vienna, Währinger Gürtel 18-20, 1090 Vienna, Austria; 2 Department of Biomedical Sciences, Humanitas University, Milan, Italy; 3 Department of Urology, IRCCS Humanitas Research Hospital, Milan, Italy; 4 Department of Urology, AOU Città della Salute e della Scienza, Torino School of Medicine, Turin, Italy; 5 Rabin Medical Center (Belenson, Hasharon), Petah Tikva, Israel; 6 Faculty of Medicine, Tel Aviv University, Tel Aviv, Israel; 7 Department of Urology, Bichat Claude Bernard Hospital, Paris Cité University, Paris, France; 8 Department of Urology and Andrology, Paracelsius Medical University, Salzburg, Austria; 9 Department of Urology, University of Cologne, Cologne, Germany; 10 Department of Pathology, Medical University of Vienna, Vienna, Austria; 11 Department of Urology, University of Texas Southwestern, Dallas, TX USA; 12 Department of Urology, Second Faculty of Medicine, Charles University, Prague, Czech Republic; 13 Division of Urology, Department of Special Surgery, The University of Jordan, Amman, Jordan; 14 Karl Landsteiner Institute of Urology and Andrology, Vienna, Austria; 15 Urology Department, Research Center for Evidence Medicine, Tabriz University of Medical Sciences, Tabriz, Iran; 16 Department of Urology, Weill Cornell Medical College, New York, NY USA; 17 Department of Urology, Semmelweis University, Budapest, Hungary

**Keywords:** Bladder cancer, En bloc, Resection, Diagnosis, Treatment, Energy source

## Abstract

**Purpose:**

To comprehensively evaluate the efficacy of different energy sources used for en-bloc transurethral resection of bladder tumors (ERBT) on perioperative outcomes.

**Methods:**

This sub-analysis derived from a prospective randomized study that enrolled patients undergoing ERBT vs conventional transurethral resection of the bladder (cTURB) from January 2019 to January 2022 (NCT03718754). Endpoints were pathological specimen quality and perioperative outcomes after either monopolar (m-ERBT) or bipolar (b-ERBT) or laser (l-ERBT) ERBT.

**Results:**

237 bladder tumors resected in 188 patients included in the analyses: 29 (12.2%) m-ERBT, 136 (57.4%) b-ERBT and 72 (30.4%) l-ERBT. Detrusor muscle (DM) was detected in 191 (80.6%) specimens. Per-tumor analysis revealed comparable rate of DM in the specimens obtained via different energy modalities (p = 0.7). Operative time was longer in the l-ERBT cohort compared to m-ERBT and b-ERBT (p = 0.02) and no obturator nerve reflex (ONR) onset was reported. On logistic regression analysis, b-ERBT was associated with negative lateral resection margins (OR 2.81; 95% CI 1.02–7.70; p = 0.04). There was no significant association of the resection technique with perforation and conversion rates (all p > 0.05). Within a median follow up of 22mo (IQR 11–29), a total of 35 (18.6%) patients had a local recurrence. On Cox regression analysis, patients resected with b-ERBT were less likely to have a recurrence (HR 0.34; 95% CI 0.15–0.78; p = 0.01); When adjusting for established confounders, this association was confirmed (HR 0.24; 95% CI 0.10–0.60; p = 0.002).

**Conclusions:**

Different energy sources might achieve comparable perioperative outcomes. Further perspectives involve the assessment of long-term differential oncological outcomes associated with various energy modalities.

**Supplementary Information:**

The online version contains supplementary material available at 10.1007/s00345-025-05565-w.

## Introduction

Transurethral resection of bladder tumor (TURB) is the first step in the treatment of urinary bladder cancer (UBC) [1].

Its main purpose is to radically remove UBC and ensure a quality sample for pathological analysis, which allows for accurate diagnosis and guides treatment and follow-up.

The quality of TURB has a major impact on patient’s own prognosis, subsequent treatments as well as total costs associated with the follow-up of UBC [2].

The presence of the detrusor muscle (DM) in the surgical specimen is a surrogate parameter for the quality of the resection and a significant prognostic factor [3].

Unfortunately, the current bulk of evidence shows that the DM is present in roughly 50% of patients who undergo TURB, leading to possible understaging and, potentially, disease progression [4]. These low rates can be partially attributed to the resection technique itself. The conventional TURB (cTURB) has several limitations which include fragmentation and thermic alterations of the specimen. This might lead to difficulties in orientation and final diagnosis during pathological examination. To improve the limitations of cTURB, the en-bloc resection of bladder tumor (ERBT) has emerged as increasingly applied technique [5–7].

This approach does not only follow the oncological principle of removing malignant tissue 'en bloc' while ensuring negative resection margin around the resected area but also allows for precise orientation and integrity of the specimen, improving the precision of histopathological analysis and staging compared to cTURB [8].

ERBT can be performed using different energy modalities [9, 10]. However, there is limited evidence investigating the differential quality of the resection as well as outcomes dependent on the energy source used. To fill this gap in knowledge, we performed a secondary analysis of a multicentric randomized controlled trial evaluating the association of different energies used during ERBT with pathological findings and perioperative outcomes.

## Materials and methods

### Study design and population

This sub-analysis forms part of a prospective, multicentric, randomized study that recruited patients between January 2019 and January 2022 (ClinicalTrials.gov Identifier: NCT03718754). The original study compared the outcomes of endoscopic resection by ERBT versus cTURB across nine European referral centers.

Inclusion criteria for the trial were patients with a cystoscopic diagnosis of primary papillary non-muscle invasive bladder carcinoma (stages cTa or cT1), tumor diameter between 1 and 3 cm, a maximum of three lesions, absence of distant metastases as confirmed by imaging, and no concurrent upper tract urothelial cancer.

For the purposes of this sub-analysis, we included only those patients who were treated with an ERBT.

### Surgical technique

Participants in the ERBT cohort underwent bladder resections employing different energy modalities contingent upon the instrumentation each participating center had and the preference of the operating surgeon and included Monopolar-ERBT (m-ERBT), Bipolar-ERBT (b-ERBT), and Laser-ERBT (l-ERBT). An enhanced visualization such as photodynamic diagnosis (PDD), narrowband imaging, or IMAGE1 S technology (previously known as SPIES) was mandatory. Typically, a circular incision was made around the tumor, maintaining a margin of approximately 5–10 mm from the neoplasm's periphery. Subsequently, the tumor was delicately dissected from the subjacent stroma, adhering meticulously to the circumscribed incisional margin.

Operation reports were homogenized across centers using a pre-established eight-item checklist [12]. No supplementary biopsies were taken from the tumor base. Perforation was documented and characterized by resection depth reaching the perivesical fat or beyond, without the need for radiographic corroboration. Postoperatively, a transurethral catheter was inserted, and bladder irrigation commenced. Additionally, a single dose of intravesical chemotherapy post-surgery was administered at the surgeon’s discretion, guided by intraoperative findings.

### Pathological evaluation and adverse event reporting

Pathological assessments were gathered across centers to include specific parameters in the pathological report: pathological stage, pathological grade (according to WHO 1973 and WHO 2004 classifications), presence or absence of DM, depth and breadth of resection margins, variant histology, lymphovascular invasion, and concomitant presence of carcinoma in situ (CIS).

Adverse events (AEs) were systematically reported utilizing the Common Terminology Criteria for Adverse Events (CTCAE).

### Outcomes

#### Primary endpoint

Primary endpoint was the quality of the pathological specimens obtained via m-ERBT, b-ERBT, or l-ERBT resections. Quality was primarily gauged by the presence of DM in the specimens, which is a widely recognized benchmark in pathological assessments of bladder resections [3, 11, 12].

#### Secondary outcomes

Secondary outcomes included: status of deep and lateral resection margins; duration of the operation; occurrence of obturator nerve reflex (ONR); necessity for conversion to conventional TURB; bladder perforation rate; incidence of post-operative complications, recurrence rate.

For data collection we used a systematic submission process to the trial unit by the investigators at each participating center, utilizing a web-based electronic data capture system (http://clincase.com). This allowed for real-time, secure, and verifiable data entry, which is essential for the integrity of multi-center trials.

### Statistical analyses

For the primary outcome we fitted logistic regression models to investigate the association of energy sources with the presence of DM in the specimen and status of deep and lateral resection margins on a per-tumor analysis. Similarly, linear and logistic regression analyses were used to investigate secondary outcomes on a per-tumor and per-patients analysis, respectively. Finally, Cox regression analyses were used to investigate the association of energy sources with time to recurrence. The survival function was visually plotted using the Kaplan–Meier method. p-values < 0.05 were considered indicative of statistical significance. Statistical analyses were conducted with Stata 17 (StataCorp LLC, College Station, TX, USA).

## Results

237 UBC resected with ERBT in 188 patients included in the analyses. 29 (12.2%) were resected with m-ERBT, 136 (57.4%) with b-ERBT, and 72 (30.4%) with l-ERBT. The clinical and pathological characteristics of the study population, stratified by energy source, are shown in Table 1.

**Table 1:** Clinical and pathologic characteristics of 188 patients treated with ERBT for primary urinary bladder cancer, stratified by energy source

Variable	b-ERBT	l-ERBT	m-ERBT	p-value
Number of patients (n)	n = 107	n = 56	n = 25	
Age (median, IQR)	68 (57–73)	60 (53.5–69.5)	64 (59–70)	0.03
Gender, n (%)				
Female	22 (20%)	21 (37.5%)	5 (20%)	0.09
Male	85 (80%)	35 (62.5%)	20 (80%)	
BMI (median, IQR)	26.12 (23.4–28.7)	25.835 (23.8–29.6)	25.3 (23–28)	0.7
Smoking status, n (%)				
Current smoker	35 (33%)	10 (18%)	10 (40%)	< 0.001
Former smoker	31 (29%)	4 (7%)	9 (36%)	
Never smoker	41 (38%)	42 (75%)	6 (24%)	
ASA (median, IQR)	2 (1–3)	2 (1–2)	2 (2–2)	0.13
ECOG (median, IQR)	0 (0–0)	0 (0–1)	0 (0–0)	0.006
Number of tumors (median, IQR)	1 (1–1)	1 (1–1)	1 (1–1)	0.8

*b-ERBT* bipolar en-bloc transurethral resection of bladder tumor, *l-ERBT* laser en-bloc transurethral resection of bladder tumor, *m-ERBT* monopolar en-bloc transurethral resection of bladder tumor, *IQR* interquartile range, *BMI* body mass index, *ASA* American Society of Anesthesiologists classification, *ECOG* Eastern Cooperative Oncology Group performance status

Overall, DM was detected in 153 (81%) patients and in 191 (80.6%) of the UBCs resected. Specifically, DM was present in 107 (79%) of b-ERBT, 24 (83%) of m-ERBT, and 60 (83%) of l-ERBT resected specimens (p = 0.47), respectively. Peri-operative outcomes stratified by energy sources on a per-tumor analysis are presented in Table 2. Intra-operative and post-operative outcomes divided by energy sources on a per-patient analysis are presented in supplementary Table 1.

**Table 2:** Peri-operative outcomes of 237 urinary bladder cancers treated with ERBT, stratified by energy sources on a per-tumor analysis

Variable	b-ERBT	l-ERBT	m-ERBT	p-value
Number of tumors	136	72	29	–
DM, n (%)				
Absent	29 (21%)	12 (17%)	5 (17%)	0.7
Present	107 (79%)	60 (83%)	24 (83%)	
Tumor location, n (%)				
Anterior wall	12 (9%)	3 (4%)	0	0.015
Left wall	41 (30%)	28 (39%)	10 (34.5%)	
Posterior wall	17 (12.5%)	15 (21%)	4 (14%)	
Right wall	34 (25%)	16 (22%)	14 (48%)	
Trigonum	32 (23.5%)	10 (14%)	1 (3.5%)	
Tumor size (median, IQR)	1.5 (1–2)	1.75 (1–2.5)	2 (1–2.5)	0.5
Deep resection margins, n (%)				
Negative	105 (77%)	42 (58%)	10 (34.5%)	< 0.001
Positive	0	0	0	
Not-evaluable	31 (23%)	30 (42%)	19 (65.5%)	
Lateral resection margins, n (%)				
Negative	92 (67%)	34 (47%)	10 (34.5%)	< 0.001
Not-evaluable	40 (30%)	34 (47%)	19 (65.5%)	
Positive	4 (3%)	4 (6%)	0	

*b-ERBT* bipolar en-bloc transurethral resection of bladder tumor, *l-ERBT* laser en-bloc transurethral resection of bladder tumor, *m-ERBT* monopolar en-bloc transurethral resection of bladder tumor, *IQR* interquartile range, *DM* detrusor muscle

On univariable logistic regression analysis there was no association between energy source and presence of DM in the specimens (p > 0.6). A statistically significant association of b-ERBT with negative lateral resection margins (OR 2.81; 95% CI 1.02–7.70; p = 0.04) was found (Supplementary Table 3). Logistic regression evaluating the association of energy modalities with deep resection margins status was not applicable due to a lack of variability, as the deep resection margins were negative in all energy groups (Table 2).

The median operative time was 26.5 (IQR 20–39) minutes and was similar across the different energy sources used for resection (p = 0.09) (supplementary Table 1). On linear regression analysis, a significant association of l-ERBT with longer operative time was found (p = 0.02; Supplementary Table 4). No ONR onset were registered in the l-ERBT group (Supplementary Table 1). Furthermore, we observed a higher rate of ONR onset in cases of tumors located on the left lateral wall [n = 11(17%); p = 0.05; Supplementary Table 2]. The rates of conversion to cTURB and perforation were comparable between groups (Supplementary Table 1). Logistic regression analysis did not show an association of energy source with these outcomes (Supplementary Table 3).

Overall, we recorded 9 (5%) cases of grade 2 complications and one grade 3 complication. There was no association of energy source with intraoperative complications (supplementary Table 3).

**Fig. 1:**
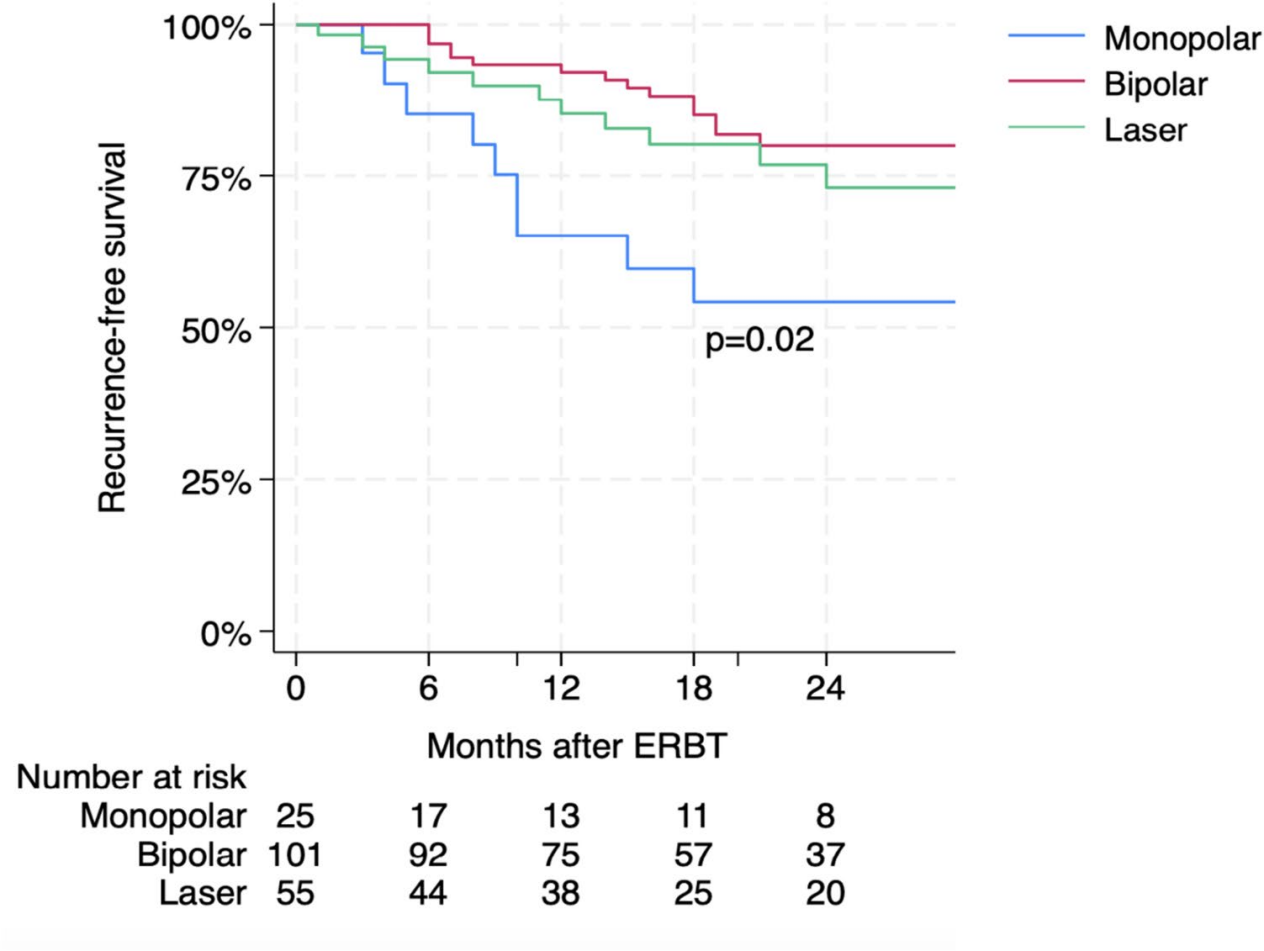
Kaplan Meier estimate of recurrence-free survival in 181 patients treated with ERBT for primary non-muscle invasive bladder cancer, stratified by energy source

Within a median follow up of 22 (IQR 11–29) months, 35 (18.6%) patients recurred. Median time to recurrence was 18 (IQR 9–27) months. On univariable cox regression analysis investigating the association with disease recurrence, a statistically significant association for bipolar energy was found (HR 0.34; 95% CI 0.15–0.78; p = 0.01). When adjusting for established confounders such as tumor pathological grade (WHO 2004), early instillation, adjuvant therapy, second-look TURB, this association was confirmed (HR 0.24; 95% CI 0.10–0.60; p = 0.002). Another important finding at multivariable cox regression analysis was represented by the association of early adjuvant instillation after TURB with recurrence (HR 0.40; 95% CI 0.18–0.93; p = 0.03). While, for pathologic grading, second-look transurethral bladder resection and adjuvant instillation therapy no association was found at univariate and multivariate Cox regression analyses (Supplementary Table 5). The Kaplan–Meier curves show visually the survival function for the energy modalities (Fig. 1) with a statistically significant difference as log-rank test revealed (p = 0.02).

## Discussion

In this post hoc analysis of the eBLOC trial (NCT03718754), we investigated the association of different energy sources used for ERBT with the quality of the pathologic specimens and found no difference between resection techniques. Current evidence supports the presence of DM in the histopathological specimen as a surrogate marker for the quality of the resection [13]. Its presence allows for proper staging, accurate risk stratification, reduces the rate of second look procedures, and ensures better outcomes [3, 11, 12]. ERBT has shown to be superior to conventional piecemeal resection in prospective and randomized trials by achieving DM rates of > 80% [5, 6, 14]. However, there is only scarce evidence on the association of different energy sources used for ERBT with the quality of the resection and perioperative outcomes. A recent secondary analysis of a single center randomized controlled trial comparing cTURB with ERBT showed no association of the energy source used for the resection with the presence of DM in the specimen [15]. The results of our analysis are in line with those reported in this study. Moreover, we expand upon these finding by adding robust data from a multicenter randomized trial to the current literature evidence.

We found a higher proportion of negative resection margins in specimens resected with b-ERBT and l-ERBT compared to those resected with m-ERBT. We acknowledge that the lack of a central pathological review might have impacted the histopathological results. Indeed, although a dedicated uropathologist at each center performed the histological analysis, the majority of m-ERBTs (72%) were performed at a single center, introducing a significant selection bias.

We found a longer operative time for resections performed with l-ERBT. This generates the hypothesis that this procedure might require longer operative time also when adopted in daily routine. This information might be helpful in daily practice for surgery scheduling.

Moreover, as previously described in retrospective series, we observe no ONR jerk during l-ERBT. This technique is, therefore, particularly suitable for the resections of tumors located on the lateral walls [16–18].

Regarding complications, we found no association of energy source used and the rate of conversion to cTURB or perforation. We report an overall perforation rate of 5.3% without significant correlation with different energy sources. The overall low rate of conversion and perforation is in line with that reported by the general literature [15, 19–21].

Current literature addresses the tumor location as one limitations of ERBT, especially in tumors located at the anterior wall or bladder dome [16]. Our findings reject this hypothesis. Location was not a limitation in patient’s selection in the trial and we could not find an association of complications or higher quality specimen with the tumor location itself.

On survival analyses, we found a significant association of b-ERBT with recurrence on univariable and multivariable cox regression analysis. When interpreting these data, one must be aware that most patients in this trial were resected with b-ERBT and most tumors included in this cohort were low-grade and low risk, introducing a significant sampling bias.

Based on current bulk evidence, ERBT does not seem to have an advantage in terms of recurrence-free survival compared to cTURB [20]. This might be associated with tumor manipulation and cell spillage despite an en-bloc resection, adjuvant instillation therapies and the tumors’ own biology itself. However, the comparative effectiveness of different energy modalities is under investigated. Further research with long-term follow-up data are needed to fill this gap in knowledge.

Our study possesses several limitations that warrant discussion. Firstly, as a secondary analysis of a randomized controlled trial, the original study was not powered to assess the outcomes measured in our analysis. Consequently, our findings should be regarded as preliminary and hypothesis-generating rather than confirmatory. Future research, designed with adequate power to investigate these specific outcomes, is needed to validate our results.

Secondly, we observed an imbalance in the utilization of energy modalities, with b-ERBT being disproportionately represented. This uneven distribution complicates any direct comparison between modalities. To mitigate this limitation, subsequent studies could be structured to ensure a more balanced application of various energy modalities.

Thirdly, the distribution of resection techniques was not uniform across participating centers, attributable to institutional resource availability and surgeons' preferences. This resulted in a center-specific predominance of certain techniques, which may introduce bias. Multicenter studies where techniques are standardized, or the effects of different institutional practices are statistically adjusted, could provide a clearer understanding of the efficacy of these techniques.

Fourthly, despite the assessment of specimens by a dedicated uropathologist, the absence of a centralized pathological review presents a limitation. While we posit that this did not substantially affect the outcomes of our analysis, the potential for inter-observer variability cannot be entirely discounted. Future studies could benefit from a multi-pathologist consensus review to enhance the reliability of pathological findings.

Finally, the follow-up was limited, restricting our ability to perform time-dependent analyses that could provide more insightful data on long-term outcomes. Longer follow-up, in future studies, would be instrumental in evaluating the sustained impact of the resection techniques on patient prognosis.

## Conclusion

This secondary analysis of the eBLOC trial generates the hypothesis that different energy sources might achieve comparable perioperative outcomes. Laser energy reduces the rate of ONR onset, might being the best choice for lesions located on the lateral walls, at the cost of longer operative times. Further perspective could involve the assessment of long-term differential oncological outcomes associated with various energy modalities.

## Supplementary Information

Below is the link to the electronic supplementary material.

Supplementary file1 (DOCX 18 KB)

Supplementary file2 (DOCX 19 KB)

Supplementary file3 (DOCX 16 KB)

Supplementary file4 (DOCX 15 KB)

Supplementary file5 (DOCX 16 KB)

## Data Availability

Data cannot be shared openly to protect study participants privacy. Data are available on request to the corresponding author.

## Abbreviations

UBC: Urinary bladder cancer
ERBT: En-bloc resection of bladder tumor
b-ERBT: ERBT-bipolar energy
m-ERBT: ERBT-monopolar energy
l-ERBT: ERBT-thulium laser energy
cTURB: Conventional transurethral resection of the bladder
ONR: Obturator nerve reflex
OR: Odds ratio
HR: Hazard ratio
CI: Confidence interval
