# Supplementary material for: Association of energy source with outcomes in en bloc TURB: secondary analysis of a randomized trial
Source: World J Urol. 2025 Mar 27;43(1):191. doi: 10.1007/s00345-025-05565-w (PMC11950035; doi:10.1007/s00345-025-05565-w)
Supplement: Supplementary file 1 — Supplementary file1 (DOCX 18 KB) [file 345_2025_5565_MOESM1_ESM.docx]

Supplementary Table 1 Perioperative outcomes of 188 patients treated with ERBT for primary non-muscle invasive bladder cancer, stratified by energy sources

|  |  | **b-ERBT** | **l-ERBT** | **m-ERBT** | **p-value** |
| --- | --- | --- | --- | --- | --- |
| Number of patients |  | N=107 | N=56 | N=25 |  |
| Operative time (median, IQR) | | 25.5 (19-32) | 30 (20-47) | 25 (20-33) | 0.09 |
| Irrigation, n (%) | No | 33 (31%) | 33 (59%) | 7 (28%) | 0.02 |
|  | Yes | 73 (68%) | 23 (41%) | 18 (72%) |  |
|  | Missing | 1 ( 1%) | 0 ( 0%) | 0 ( 0%) |  |
| Early instillation^1^, n (%) | No | 73 (68%) | 10 (18%) | 10 (40%) | <0.001 |
|  | Yes | 33 (31%) | 46 (82%) | 15 (60%) |  |
|  | Missing | 1 ( 1%) | 0 ( 0%) | 0 ( 0%) |  |
| Concomitant CIS, n (%) | No | 101 (94%) | 53 (95%) | 24 (96%) | 0.9 |
|  | Yes | 5 ( 5%) | 3 ( 5%) | 1 ( 4%) |  |
|  | Missing | 1 ( 1%) | 0 ( 0%) | 0 ( 0%) |  |
| T1 substage feasibility, n (%) | | 16 (15%) | 15 (27%) | 3 (12%) | 0.2 |
| NMIBC, n (%) | HG | 37 (35%) | 11 (20%) | 6 (24%) | 0.02 |
|  | LG | 63 (58.5%) | 39 (69%) | 18 (72%) |  |
|  | Missing | 7 ( 6.5%) | 6 (11%) | 1 ( 4%) |  |
| Conversion to cTURBT, n (%) | No | 105 (98%) | 53 (95%) | 24 (96%) | 0.7 |
|  | Yes | 2 ( 2%) | 3 ( 5%) | 1 ( 4%) |  |
| ONR onset, n (%) | No | 96 (90%) | 56 (100%) | 19 (76%) | 0.03 |
|  | Yes | 10 ( 9%) | 0 ( 0%) | 6 (24%) |  |
|  | Missing | 1 ( 1%) | 0 ( 0%) | 0 ( 0%) |  |
| CTCAE, n (%) | 2 | 5 ( 5%) | 3 ( 5%) | 1 ( 4%) | 0.8 |
|  | 3 | 0 ( 0%) | 1 ( 2%) | 0 ( 0%) |  |
|  | No | 102 (95%) | 52 (93%) | 24 (96%) |  |
| reTURB, n (%) | No | 86 (80%) | 53 (95%) | 21 (84%) | 0.4 |
|  | Yes | 20 (19%) | 3 ( 5%) | 4 (16%) |  |
|  | Missing | 1 ( 1%) | 0 ( 0%) | 0 ( 0%) |  |
| Perforation, n (%) | No | 101 (94%) | 52 (93%) | 24 (96%) | 0.9 |
|  | Yes | 5 ( 5%) | 4 ( 7%) | 1 ( 4%) |  |
|  | Missing | 1 ( 1%) | 0 ( 0%) | 0 ( 0%) |  |
| Surgical experience, n (%) | consultant <5y | 28 (26%) | 15 (27%) | 12 (48%) | <0.001 |
|  | consultant >5y | 60 (56%) | 38 (68%) | 3 (12%) |  |
|  | Resident | 19 (18%) | 3 ( 5%) | 10 (40%) |  |

Supplementary Table 1.. Abbreviations: b-ERBT= bipolar en-bloc transurethral resection of bladder tumor; l-ERBT= laser en-bloc transurethral resection of bladder tumor; m-ERBT= monopolar en-bloc transurethral resection of bladder tumor; IQR=interquartile range; CIS=Carcinoma In Situ; cTURB=conventional Transurethral Resection of Blabber tumor; NMIBC=Non-muscle invasive bladder cancer; LG= Low-grade; HG=High grade; ONR= Obturator Nerve Reflex; CTCAE=Common Terminology Criteria for Adverse Events; DM=Detrusor Muscle;

*^1^ Early intravesical instillation after TURB*
