# Supplementary material for: Association of energy source with outcomes in en bloc TURB: secondary analysis of a randomized trial
Source: World J Urol. 2025 Mar 27;43(1):191. doi: 10.1007/s00345-025-05565-w (PMC11950035; doi:10.1007/s00345-025-05565-w)
Supplement: Supplementary file 2 — Supplementary file2 (DOCX 19 KB) [file 345_2025_5565_MOESM2_ESM.docx]

*Supplementary Table 2. Perioperative outcomes of 188 patients treated with ERBT for primary non-muscle invasive bladder cancer, stratified by tumor location*

|  |  | **Anterior wall** | **Left wall** | **Posterior wall** | **Right wall** | **Trigonum** | **p-value** |
| --- | --- | --- | --- | --- | --- | --- | --- |
| Number of patients (N) | | N=9 | N=65 | N=31 | N=50 | N=33 |  |
| Operative time (median,IQR) | | 30  (20-36) | 26  (20-40) | 30  (20-45) | 24.5  (15-30) | 29  (20-38) | 0.4 |
| Irrigation, n (%) | No | 2 (22%) | 27 (42%) | 16 (53%) | 23 (46%) | 5 (15%) | 0.010 |
|  | Yes | 7 (78%) | 38 (58%) | 14 (47%) | 27 (54%) | 28 (85%) |  |
| Early instillation^1^, n (%) | No | 7 (78%) | 25 (38%) | 15 (50%) | 25 (50%) | 21 (65%) | 0.06 |
|  | Yes | 2 (22%) | 40 (62%) | 15 (50%) | 25 (50%) | 12 (35%) |  |
| Concomitant CIS, n (%) | No | 9 (100%) | 62 (95%) | 26 (87%) | 49 (98%) | 32 (97%) | 0.18 |
|  | Yes | 0 (0%) | 3 (5%) | 4 (13%) | 1 (2%) | 1 (3%) |  |
| T1 substage feasibility, n (%) | | 1 (11%) | 17 (26%) | 5 (17%) | 8 (16%) | 3 (9%) | 0.09 |
| NMIBC, n (%) | HG | 4 (44%) | 19 (29%) | 9 (30%) | 15 (30%) | 6 (20%) | 0.25 |
|  | LG | 5 (55%) | 46 (71%) | 17 (56%) | 29 (58%) | 23 (68%) |  |
|  | Missing | 0 (0%) | 0 (0%) | 4 (13%) | 6 (12%) | 4 (12%) |  |
| Conversion to cTURBT, n (%) | No | 8 (89%) | 62 (95%) | 29 (97%) | 50 (100%) | 32 (97%) | 0.4 |
|  | Yes | 1 (11%) | 3 (5%) | 1 (3%) | 0 (0%) | 1 (3%) |  |
| ONR onset, n (%) | No | 9 (100%) | 54 (83%) | 29 (97%) | 48 (96%) | 31 (94%) | 0.05 |
|  | Yes | 0 (0%) | 11 (17%) | 1 (3%) | 2 (4%) | 2 (6%) |  |
| CTCAE, n (%) | 2 | 0 (0%) | 3 (5%) | 0 (0%) | 3 (6%) | 3 (9%) | 0.4 |
|  | 3 | 0 (0%) | 0 (0%) | 1 (3%) | 0 (0%) | 0 (0%) |  |
|  | No | 9 (100%) | 62 (95%) | 29 (97%) | 47 (94%) | 30 (91%) |  |
| Energy source, n(%) | b-ERBT | 8 (89%) | 33 (51%) | 15 (50%) | 24 (48%) | 25 (76%) | 0.02 |
|  | l-ERBT | 1 (11%) | 23 (35%) | 12 (40%) | 12 (24%) | 8 (24%) |  |
|  | m-ERBT | 0 (0%) | 9 (14%) | 3 (10%) | 13 (26%) | 0 (0%) |  |
| reTURB, n (%) | No | 8 (89%) | 50 (77%) | 26 (87%) | 47 (94%) | 29 (88%) | 0.13 |
|  | Yes | 1 (11%) | 15 (23%) | 4 (13%) | 3 (6%) | 4 (12%) |  |
| Perforation, n (%) | No | 9 (100%) | 62 (95%) | 29 (97%) | 47 (94%) | 30 (91%) | 0.8 |
|  | Yes | 0 (0%) | 3 (5%) | 1 (3%) | 3 (6%) | 3 (9%) |  |
| Surgical experience, n (%) | consultant <5y | 1 (11%) | 23 (35%) | 5 (17%) | 14 (28%) | 12 (35%) | 0.40 |
|  | consultant >5y | 6 (67%) | 31 (48%) | 22 (73%) | 26 (52%) | 15 (47%) |  |
|  | Resident | 2 (22%) | 11 (17%) | 3 (10%) | 10 (20%) | 6 (18%) |  |

Supplementary Table 2. Abbreviations: IQR=interquartile range; CIS=Carcinoma In Situ; cTURB=conventional Transurethral Resection of Blabber tumor; NMIBC=Non-muscle invasive bladder cancer; LG= Low-grade; HG=High grade; ONR=Obturator Nerve Reflex; CTCAE=Common Terminology Criteria for Adverse Events; DM=Detrusor Muscle;

^1^ Early intravesical instillation after TURB.
