# Supplementary material for: Association of energy source with outcomes in en bloc TURB: secondary analysis of a randomized trial
Source: World J Urol. 2025 Mar 27;43(1):191. doi: 10.1007/s00345-025-05565-w (PMC11950035; doi:10.1007/s00345-025-05565-w)
Supplement: Supplementary file 3 — Supplementary file3 (DOCX 16 KB) [file 345_2025_5565_MOESM3_ESM.docx]

Supplementary Table 3. Logistic Regression analysis investigating the association of energy source with perioperative outcomes in 188 patients treated with ERBT for primary non-muscle invasive bladder cancer

|  | **DM^1^** | **Deep resection margin^1,2^** | **Negative lateral resection margin^1,3^** | **ONR onset ^4,5^** | **Conversion to cTURB^4^** | **Perforation^4^** | **CTCAE ^4,6^** |
| --- | --- | --- | --- | --- | --- | --- | --- |
| **m-ERBT OR^7^;95%CI^8^; p-value** | - | - | - | - | - | - | - |
| **b-ERBT OR^7^; 95%CI^8^; p-value** | 0.77;0.27-2.21;0.6 | - | 2.81;1.02-7.70;**0.04** | 0.32;0.10-1.00;0.051 | 0.45;0.04-5.25;0.53 | 1.17;0.13-10.53;0.9 | 1.17;0.13-10.53;0.9 |
| **l-ERBT OR^7^;95%CI^8^;p-value** | 1.04;0.33-3.27;0.9 | - | - | - | 1.36;0.13-13.74;0.8 | 1.84;0.19-17.41;0.6 | 1.84;0.19-17.41;0.6 |

Supplementary Table 3.

^1^Per-tumor analysis;^2^Logistic regression not possible for absence of variability (negative is the only value for deep resection margin);^3^ no variability in the m-ERBT cohort ( all lateral resection margins were negative) and collinearity in the l-ERBT group;^4^per-patient analysis; ^5^Patients treated with l-ERBT were removed in this analysis for the absence of events in this cohort; ^6^CTCAE grade 2 and 3 were merged, thus considering in this analysis the presence or absence of CTCAE.

*^7^Odds ratio; ^8^Confidence Interval*

Abbreviations: DM= Detrusor Muscle; ONR=Obturator Nerve Reflex; cTURB=conventional Transurethral Resection of the Bladder; CTCAE=Common Terminology Criteria for Adverse Events
