# Supplementary material for: Association of energy source with outcomes in en bloc TURB: secondary analysis of a randomized trial
Source: World J Urol. 2025 Mar 27;43(1):191. doi: 10.1007/s00345-025-05565-w (PMC11950035; doi:10.1007/s00345-025-05565-w)
Supplement: Supplementary file 4 — Supplementary file4 (DOCX 15 KB) [file 345_2025_5565_MOESM4_ESM.docx]

*Supplementary Table 4. Linear Regression analysis investigating the association of energy source with operative time in 188 patients treated with ERBT for primary non-muscle invasive bladder cancer*

| **Operative time** |  | **Coefficient** | **Std.error** | **t** | **P> \|t\|** | **95%CI** |
| --- | --- | --- | --- | --- | --- | --- |
| **Energy source** | monopolar | - | - | - | - | - |
|  | bipolar | 2.85 | 3.85 | 0.74 | 0.46 | -4.75-10.47 |
|  | laser | 9.60 | 4.17 | 2.30 | **0.02** | 1.37-17.84 |
| _cons | | 25.68 | 3.47 | 7.40 | 0.000 | 18.83-32.52 |

*Supplementary Table 4.*
