# Supplementary material for: Association of energy source with outcomes in en bloc TURB: secondary analysis of a randomized trial
Source: World J Urol. 2025 Mar 27;43(1):191. doi: 10.1007/s00345-025-05565-w (PMC11950035; doi:10.1007/s00345-025-05565-w)
Supplement: Supplementary file 5 — Supplementary file5 (DOCX 16 KB) [file 345_2025_5565_MOESM5_ESM.docx]

Supplementary Table 5. Univariable & Multivariable Cox regression analyses investigating the association of energy source with recurrence in 188 patients treated with ERBT for primary non-muscle invasive bladder cancer

| Variable | **Univariable** | | | **Multivariable** | | |
| --- | --- | --- | --- | --- | --- | --- |
|  | **HR^1^** | **95%CI^2^** | **p^3^** | **HR^1^** | **95%CI^2^** | **p^3^** |
| Energy source  monopolar  bipolar  laser | -  0.34  0.43 | -  0.15-0.78  0.17-1.06 | **-**  **0.01**  0.07 | -  0.24  0.50 | -  0.10-0.60  0.18-1.36 | **-**  **0.002**  0.17 |
| NMIBC  LG  HG | -  0.64 | -  0.30-1.37 | -  0.25 | -  0.62 | -  0.24-1.58 | -  0.32 |
| Early instillation^4^ | 0.80 | 0.40-1.55 | 0.5 | 0.40 | 0.18-0.93 | **0.03** |
| Adjuvant instillation  None  BCG  Chemotherapy agent | -  0.82  1.19 | -  0.28-2.40  0.56-2.54 | -  0.72  0.64 | -  0.73  1.28 | -  0.19-2.80  0.52-3.17 | -  0.65  0.60 |
| ReTURB | 1.06 | 0.41-2.75 | 0.9 | 2.22 | 0.68-7.25 | 0.19 |

Supplementary Table 5. Abbreviations:NMIBC= non-muscle invasive bladder cancer; LG=low grade; HG=High grade; BCG= Bacillus of Calmette-Guèrin; TURB= Transurethral resection of the bladder.

^1^Hazard ratio; ^2^Confidence interval; ^3^p-value; ^4^Early intravesical instillation after TURB
